# P-1800. Antimicrobial Stewardship Compliance with Accreditation Requirements within a Hospital Pharmacy Organization

**DOI:** 10.1093/ofid/ofae631.1963

**Published:** 2025-01-29

**Authors:** Katherine M Shea, Athena L V Hobbs, Steve Lundquist, Jessica Sun

**Affiliations:** Cardinal Health, Austin, Texas; Cardinal Health, Austin, Texas; Cardinal Health, Austin, Texas; Cardinal Health, Austin, Texas

## Abstract

**Background:**

Between 2022 and 2023 the Center for Medicare and Medicaid Services and The Joint Commission updated requirements and/or guidance related to antibiotic stewardship programs (ASPs). In June 2023, an antimicrobial stewardship gap analysis survey was required within all Cardinal Health pharmacy managed facilities to assess for compliance with accreditation requirements and identify areas for improvement.

**Methods:**

This was a 55 question multi-site survey assessing compliance with accreditation requirements for hospital ASPs. Participants responded to questions as yes, no, or not applicable. Compliance was defined as a “yes” or “not applicable” response. A chi-square was used to assess responses between hospital types and average daily census (ADC).

**Results:**

122 hospitals completed the survey (41 hospitals, 17 critical access, 27 long-term acute care (LTAC), 29 rehabilitation, 8 LTAC/rehabilitation). Most facilities had an ADC of < 50 (< 26: 46, 26-50: 44, 51-100: 17, 101-200: 7, 201-500: 8). The mean (+ STD) compliance within all hospitals was 81.7% (+ 5.1%). A significant difference in compliance with accreditation requirements was found based on hospital type (p< 0.001) and ADC (p< 0.001). Rehabilitation and LTAC hospitals had the highest compliance rates of 89% and 83%, respectively. When assessed by ADC, the lowest compliance rates were found in hospitals with less than 26 beds (78.8%).

The top five needs with < 75% compliance included documentation of program outcomes, documentation of evidence-based use of antibiotics in all departments and services of the hospital, reporting progress towards antibiotic stewardship goals and initiatives to hospital leadership, evaluating adherence to an evidence-based guideline, and providing competency-based education to hospital staff.

**Conclusion:**

This standardized survey identified ASP gaps based on accreditation requirements and identified key areas for improvement. Interestingly, LTACs and rehabilitation hospitals had the highest compliance rates of all facility types; however, the largest post-acute system within the pharmacy managed hospitals, which accounted for 84.4% of all LTAC and rehabilitation hospitals, has a system-level antimicrobial stewardship committee as well as a dedicated infectious diseases pharmacist.

**Disclosures:**

**Katherine M. Shea, PharmD, BCIDP**, T2 Biosystems: Advisor/Consultant

